# Investigating the Efficacy of a Canine Rabies Vaccine Following Storage Outside of the Cold-Chain in a Passive Cooling Device

**DOI:** 10.3389/fvets.2021.728271

**Published:** 2021-09-29

**Authors:** Ahmed Lugelo, Katie Hampson, Anna Czupryna, Machunde Bigambo, Lorraine M. McElhinney, Denise A. Marston, Rudovick Kazwala, Felix Lankester

**Affiliations:** ^1^Environmental Health and Ecological Sciences Department, Ifakara Health Institute, Dar es Salaam, Tanzania; ^2^Boyd Orr Centre for Population and Ecosystem Health, Institute of Biodiversity, Animal Health and Comparative Medicine, University of Glasgow, Glasgow, United Kingdom; ^3^Department of Veterinary Medicine and Public Health, Sokoine University of Agriculture, Morogoro, Tanzania; ^4^Global Animal Health Tanzania, Arusha, Tanzania; ^5^Animal and Plant Health Agency, Addlestone, United Kingdom; ^6^Paul G. Allen School for Global Health, Washington State University, Pullman, WA, United States

**Keywords:** dog-mediated rabies, mass vaccination, thermotolerance, passive cooling device, cold-chain, body condition score, vaccine storage

## Abstract

**Background:** Thermostable vaccines greatly improved the reach and impact of large-scale programmes to eliminate infectious diseases such as smallpox, polio, and rinderpest. A study from 2015 demonstrated that the potency of the Nobivac^®^ Rabies vaccine was not impacted following experimental storage at 30°C for 3 months. Whether the vaccine would remain efficacious following storage under more natural, fluctuating temperature conditions remains unknown. We carried out a randomised controlled non-inferiority trial to compare serological responses in dogs following vaccination with doses stored under cold chain conditions with those stored within a locally made Passive Cooling Device (“Zeepot”) under fluctuating temperature conditions.

**Materials and Methods:** Nobivac^®^ Rabies vaccine was stored under either cold-chain conditions or within the Zeepot for 2 months. Daily ambient temperatures and temperatures within the Zeepot were recorded every 3 h. Following storage, 412 domestic dogs were randomly assigned to receive either cold-chain or Zeepot stored Nobivac^®^ Rabies vaccine. Baseline and day 28-post vaccination blood samples were collected. Serological analysis using the Fluorescent Antibody Virus Neutralisation assay was carried out with a threshold of 0.5 IU/ml to determine seroconversion. In addition, the impact of dog Body Condition Score, sex, and age on seroconversion was examined.

**Results:** The serological response of dogs vaccinated using Nobivac^®^ Rabies vaccine stored within the Zeepot was not inferior to the response of dogs vaccinated using cold-chain stored vaccine (z = 1.1, df = 313, *p*-value = 0.25). Indeed, the 28-day post-vaccination group geometric mean titre was 1.8 and 2.0 IU/ml for cold-chain vs. non-cold-chain storage, respectively. Moreover, the percentage of dogs that seroconverted in each arm was almost identical (85%). There was a positive linear trend between Body Condition Score (O.R. 2.2, 95% CI: 1.1–5.1) and seroconversion, suggesting dogs of poor condition may not respond as expected to vaccination.

**Conclusions:** Our study demonstrated the potency of Nobivac^®^ Rabies vaccine is not impacted following storage under elevated fluctuating temperatures within a Zeepot. These results have potentially exciting applications for scaling up mass dog vaccination programmes in low-and-middle income countries, particularly for hard-to-reach populations with limited access to power and cold-chain vaccine storage.

## Introduction

Canine rabies causes ~60,000 human deaths every year worldwide, most of the victims being children under 15 years of age (1). More than 95 per cent of these fatalities occur in developing countries. Since the first rabies vaccine was developed in the 1880s by Louis Pasteur, considerable efforts have been invested in the development of high-quality rabies vaccines for humans and animals (2, 3). Epidemiological and operational research has shown that domestic dogs are the reservoir for rabies in countries where human rabies remains a concern, and that mass dog vaccination is a cost-effective way of controlling and ultimately eliminating rabies from dog and human populations. However, implementing vaccination programmes at scale requires functioning cold chain systems which are often not available in the countries where rabies is still endemic. Indeed, the high cost of installation, training of personnel, and unreliable electricity have been identified as major challenges that hinder establishment of the cold chain system in developing countries (4, 5).

Challenges of transporting and storing vaccines hinder the last mile of immunisation, and as such considerable efforts have been invested to tackle these challenges on many fronts (5–7). For example, in recent decades stakeholders in the field of public health have encouraged vaccine manufacturers to produce thermotolerant vaccines as one way of addressing the problem. As a result, a number of thermotolerant vaccines have been developed and are already available (8–11), whilst several others are at different stages of development and approval (12). For example, thermotolerant vaccines have been developed and used to effectively control smallpox, meningitis A, Newcastle disease, and rinderpest (11–13). In addition to these well-known examples, a study conducted in 2015 demonstrated that the potency of Nobivac^®^ Rabies canine vaccine was not impacted following storage under *fixed* experimental conditions at 25°C for 6 months and 30°C for 3 months (14). This later example provides hope that this rabies vaccine can be used following local storage outside of cold chain conditions in remote places where rabies remains endemic.

In addition to efforts to develop thermotolerant vaccines, innovations to develop equipment suitable for storage of vaccines in isolated areas is also ongoing (15, 16). These innovations have led to the production of a variety of off-grid equipment ranging from simple cold boxes, used to transport vaccines from the storage facility to the immunisation site, to solar-powered refrigerators (16). Further, the development of off-grid refrigerators, such as solar refrigerators, have helped to bring life-saving vaccinations to hard-to-reach populations who would otherwise be lost to vaccine preventable diseases. Despite their benefits, however, solar refrigerators are costly to establish and maintain. Additionally, solar refrigerators are sophisticated pieces of equipment and require specialist technicians, who are typically not available in remote regions of Sub-Saharan Africa and Asia, to repair problems that occur (17, 18). Thus, the use of this equipment has been constrained to a limited number of places. In response to these issues, manufacturers have directed their efforts to develop another generation of cold chain equipment known as passive cooling devices (PCDs). These devices do not need electricity to keep the internal compartments cool, instead they rely on effective insulation and cooling media to create a cool environment. For example, the Arktek^®^ passive vaccine storage device, which was used to transport and store vaccines during the 2014 Ebola outbreak in west Africa, uses dry ice for cooling and has the ability to keep the vaccine compartment at 2–10°C for 35 days. However, the high purchase price of the Arktek^®^ device which ranges from US$1,200 to US$2,400 and the lack of availability of ice in remote areas has limited the widespread utility of this tool in developing countries (19). These limitations have encouraged designers to focus their efforts into developing inexpensive and simple PCDs. One promising outcome of this challenge has been the development of the very low cost PCD called a Zeepot which is built from local materials such as clay and wood. A single Zeepot costs ~$11 and is able to keep, on average, the internal temperature at 20°C below ambient temperature (19).

Availability of thermotolerant vaccines and affordable PCDs such as the Zeepot have the potential to transform ongoing rabies control efforts in resource-poor settings. The primary aim of this study, therefore, was to investigate whether the potency of the thermotolerant Nobivac^®^ Rabies vaccine was impacted by long-term storage inside the locally made Zeepot under field conditions where temperatures naturally fluctuate. A secondary aim of the study was to investigate the impact that body condition score and age of domestic dogs have on immunogenicity. It is expected that the outcomes from this study will inform alternative cost-effective means of delivering rabies vaccination to dogs at scale in remote regions which frequently suffer from cold chain constraints.

## Materials and Methods

A controlled and randomised non-inferiority study was carried out to compare the serological response in dogs following vaccination with doses of Nobivac^®^ Rabies vaccine (MSD, Boxmeer, Netherlands) stored under either cold-chain conditions (cold-chain storage) or within the Zeepot (non-cold-chain storage). The Zeepot was made of clay and wood as previously described by Lugelo et al. (19). The Nobivac rabies vaccine is inactivated and contains ≥2 I.U. rabies virus strain Pasteur RIV per dose and thiomersal 0.01% as preservative.

### Preparation of Vaccines

Prior to the commencement of the field trial, Nobivac^®^ Rabies vaccines were stored under cold-chain or non-cold chain (Zeepot) conditions for a 2-month period. The study began 6 months after the vaccines' date of manufacture. The Zeepot was placed in the living room, which is generally in the shade. The daily temperature inside the Zeepot was collected at 3 hourly intervals using a digital temperature data logger (Sensormetrix^®^). Similarly, the ambient temperature of the storage room in which the Zeepot was kept was recorded using the same device.

### Study Location and Dog Population

The field trial was carried out in eight villages within the Serengeti district of northern Tanzania. These villages are Gesarya, Kebanchebanche, Kemgesi, Ngarawani, Nyamatare, Nyamoko, Nyamatoke, and Kwitete. Based on a complete census carried out between 2010 and 2015 as part of a research study, ~26,756 people and 6,203 dogs live in these eight villages. Dogs are kept mostly for household security, hunting and protecting livestock from predators (20, 21). Despite being owned, the vast majority of these dogs roam freely and they rarely receive veterinary care. This is a common situation in many parts of the country and other areas of rural Africa (22).

### Enrolment of Dogs

Two teams of researchers accompanied by a local community leader walked on foot to locate dog keeping households within each study village. Upon arriving at a household, the researcher with the help of the community leader explained the purpose of the study and asked the head of the household whether they were happy for their dog to participate in the study. Following the signing of Informed Consent by the owner, all unvaccinated dogs present at the household, irrespective of their body condition score (BCS) and ages, were enrolled in the trial. The BCS used a scale of 1 (emaciated) to 5 (obese). Previously vaccinated dogs were not enrolled in the study, instead they received a standard-cold chain stored vaccine.

### Sample Size Calculations

Sample size calculations were the same as those performed in the prior controlled and randomised non-inferiority themotolerance study carried out by Lankester et al. (14). In brief, the sample size for this study was estimated to be 50 dogs per group. This would give at least 80% power at the 5% significance level to detect non-inferiority of serological responses (the primary measurable output) to non-cold chain relative to cold-chain-stored vaccine, assuming a non-inferiority margin of −1.2 log^2^ titre units and a standard deviation of 1.8 log^2^ titre units. We also planned to carry out an exploratory analysis to assess the impact of BCS and age as determinants of immunogenicity. Given this exploratory analysis, and given the aim of increasing confidence in the results and the availability of large numbers of local dogs, the sample size was increased to ~200 dogs per group.

### Sample Collection and Vaccination

Prior to sample collection, each enrolled dog was registered, had key biodata collected (e.g., age, BCS, sex), and a microchip inserted into the scruff of the neck. Whole blood samples were collected from the cephalic vein in S-Monovette^®^ tubes (Sarsted AG, Nümbrecht, Germany). The blood samples were placed in a cool box for transportation to the laboratory.

### Vaccination

The vaccines were prepared in the following manner: equal quantities of Group 1 (cold-chain) and Group 2 (Zeepot) stored vaccine were loaded into syringes each day prior to commencing field activities. Dogs were randomly assigned to receive vaccine from either group. The randomisation was achieved through the random assortment of syringes loaded with vaccine and subsequent inoculation to each dog being enrolled. Vaccination was carried out through subcutaneous injection of 1 ml of Nobivac^®^ Rabies vaccine. The vaccines prepared each day were all used to ensure the number of dogs recruited in each group were equal. This process was repeated on the following day until a total of 200 dogs per group was reached. The vaccines were coded so that neither dog owners nor research staff knew the treatment given to each individual dog. Dog owners were told to contact the research team veterinarian via the telephone number written on the consent form if they observed any adverse effects that were likely attributable to the inoculation.

### Follow Up Visit

Post-vaccination blood sampling was carried out at day 28 (4 weeks) after the inoculation. The field team visited the same households, study dogs were identified via the microchip reader and blood samples were collected and stored in the same manner as at day 0. All dogs were then vaccinated with a cold-chain stored vaccine to ensure effective immunogenicity.

### Sample Processing

Following storage in the cold box and transfer to the laboratory, all blood samples were kept at room temperature for 12 h before being centrifuged to separate the serum. Aliquots of serum (1 ml) were taken from each sample and were kept at −20°C prior to shipment to the Animal and Plant Health Agency (APHA, UK) for serological analyses.

### Serological Analyses

The Fluorescent Antibody Virus Neutralisation assay (FAVN) was used to determine levels of antibody titres against rabies (23). In brief, serial 3-fold dilutions of the positive and negative serum controls as well as of the test serum were prepared in the microplates. Each serum was added to four adjacent wells and serially diluted four times. A 50-μL of a dilution of challenge virus standard (CSV-11) containing 50–200 TCID50/ml was then added to each serum dilution well. The microplates were incubated for 1-h at 37°C in a humidified incubator with 5% CO_2_. Following incubation, 50-μL of the cell suspension with concentration of 4 × 10^5^ BHK-21 cells/ml was added to each well and further incubated for 48 h at 37°C in a humidified incubator with 5% CO_2_. After incubation, the plates were fixed in 80% acetone, dried, and stained with fluorescein isothiocyanate (FITC) anti-rabies monoclonal antibody (Fujirebio Diagnostics, Malvern, PA, USA). For each serum dilution, four wells were examined for the presence or absence of virus-infected cells. The 50% endpoint of the antibody (D50) content of test sera and virus titrations (TCID_50_) were calculated according to the Spearman-Kärber method. To determine a titre, the control “cell only” wells needed a full monolayer of confluent cells >80%. If serum cytotoxicity was detected, the wells for that specific sample were not read and the sample was filtered and repeated. By international convention, this titre was converted to a value in IU/mL by comparison of results obtained with those of the positive standard. The level of rabies neutralising antibodies was expressed in International Unit per millilitre (IU/ml). A cut off value of 0.5 IU/ml was used to determine seroconversion (24).

### Statistical Analysis

Titre measurements were statistically analysed using two-sample *T*-test. *Post-hoc* estimates of the differences between elevated storage conditions and the cold-chain stored vaccine were calculated with one-sided 95% confidence intervals. The null hypothesis, of elevated storage condition being inferior to the cold-chain storage, was rejected if the difference between the groups did not exceed <1.2 log_2_ titre units. Multivariate logistic regression analysis was used to determine whether storage conditions, BCS or age of dog impacted the likelihood of seroconversion (titres ≥ 0.5 IU/ml).

## Results

### Study Population

**Figure 1:**
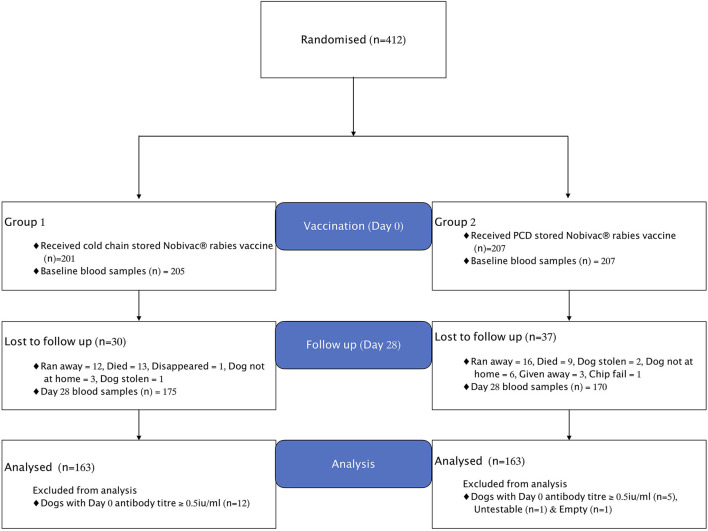
Flowchart showing enrolment, treatments, and losses of dogs in the vaccine trial. Empty means vial without sample and Untestable means sample conditions were not appropriate for testing.

The enrolment and vaccine group assigned to dogs is shown in the study flow chart (Figure 1). In total, 412 domestic dogs with no previous history of rabies vaccination were included in the study. Of these 412 dogs, 205 received cold-chain- and 207 received Zeepot-stored vaccine.

### Vaccine-Related Adverse Events

During the follow up visit at day 28, a total of 22 dogs, 13 in Group 1 and nine in Group 2 were reported to have died. Of the 22 dogs, one died suddenly without showing any clinical signs and four dogs died due to fatal injuries caused by: severe bites from other dogs (*n* = 1), bites from a hyena (*n* = 1), being beaten by the owner (*n* = 1) and being trampled by cows (*n* = 1). The remaining 17 dogs died showing signs of illness, mostly within 2 weeks of the vaccination; 12 of these dogs (71%) were <6 months in age. Clinical signs included vomiting, diarrhoea, emaciation, coughing, anorexia, nasal discharges, dullness, and incoordination. Dogs in both groups presented similar clinical signs. Further investigation revealed that dogs from households not participating in the study within and outside the study villages exhibited similar clinical signs and died, suggesting that the cause of death among the study animals was not related to immunisation. Although the symptoms were suggestive of an infectious aetiology, a definitive diagnosis was not determined. Rabies could not be ruled out in four of the dogs that died, but none showed typical signs of rabies, although post-mortem samples were not collected for testing. No rabies cases were reported in any of the study villages of these dogs in the 2 years following the study, although four rabies cases were detected in the two of the villages in the 6 months prior to the study.

### Lost to Follow Up and Post-inclusion Removal

Out of 412 dogs enrolled into the study at day 0, only 341 dogs were available at day 28 for re-sampling, with 67 lost to follow-up for the reasons shown in Figure 1. Seventeen out of 412 dogs sampled at day 0 were found to have pre-vaccination antibody titres ≥ 0.5 IU/ml and were thus excluded from the analysis. As a result, a total of 326 dogs were included in the final analyses, with 163 dogs in each treatment group as summarised in Table 1 below.

**Table 1:** Summary showing the distribution of age, sex, and BCS of dogs in the two groups included in the final analysis.

**Variable**	**Category**	**Group 1 (cold-chain stored vaccine)**	**Group 2 (Zeepot-stored vaccine)**
Age (months)			
	0–3	12	15
	3–6	44	41
	6–12	52	53
	12–24	30	32
	24–36	11	10
	36–48	10	5
	48–60	2	2
	>60	2	5
Body condition score			
	1	28	47
	2	104	88
	3	31	28
	4	0	0
	5	0	0
Sex			
	Males	75	70
	Females	88	93

### Assessment of Vaccine Storage in the Zeepot

**Figure 2:**
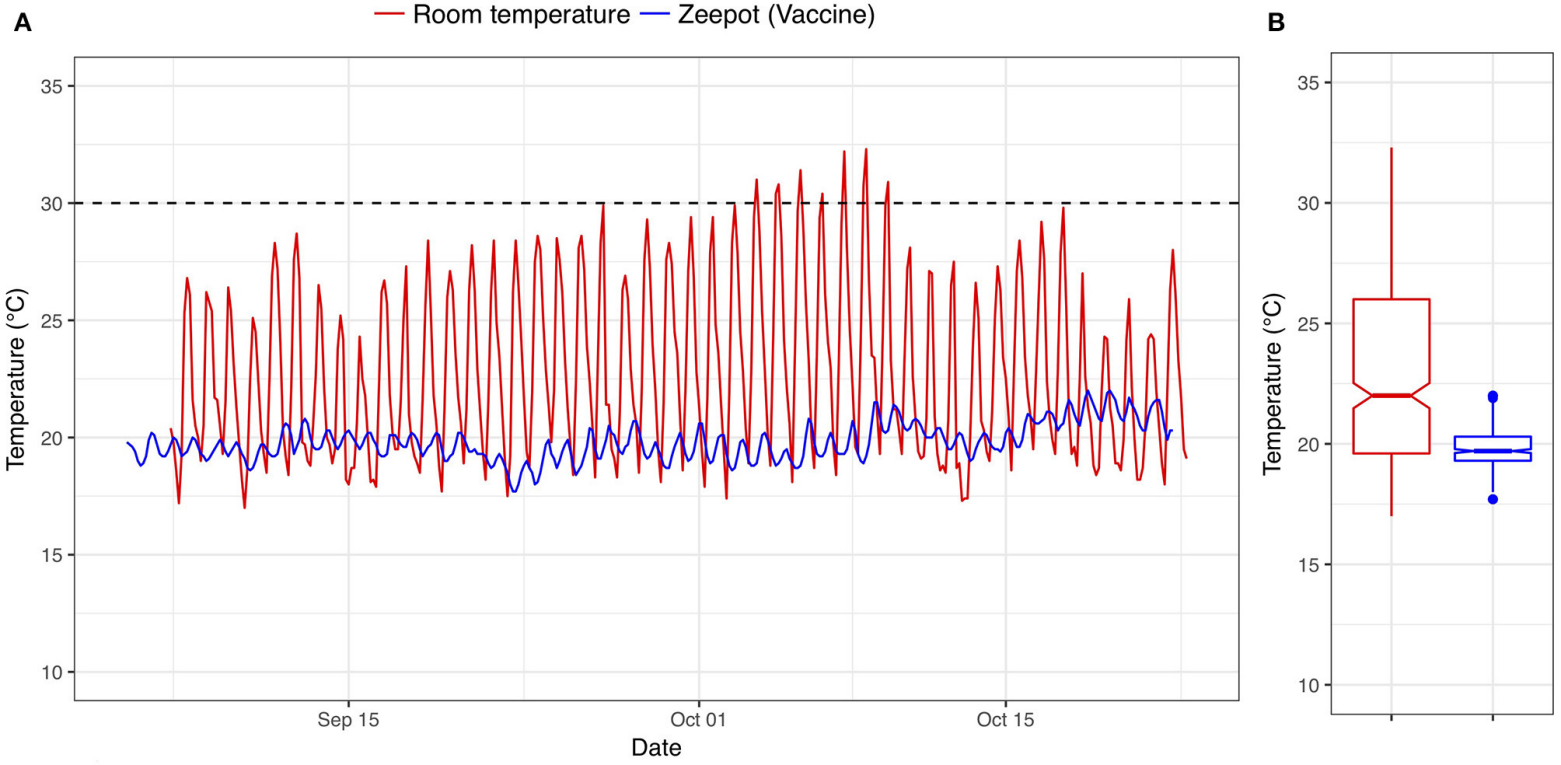
**(A,B)** Time series and boxplot showing the temperatures of vaccines stored within the Passive Cooling Device (blue line) and the ambient room temperature (red line); dashed line indicates the maximum storage temperature below which Nobivac^®^ Rabies vaccines have been shown to remain potent.

The room temperatures and the vaccine storage temperatures inside the Zeepot recorded over the 2-month storage period are shown in Figure 2. The mean room temperature was 22.84°C ranging from 17.00 to 32.2°C whereas the average temperature inside the Zeepot was 19.81°C fluctuating between 17.7 and 22°C. Despite ambient temperatures exceeding 30°C, the internal Zeepot temperature did not exceed 22°C, which was below the storage temperature at which the vaccines have been shown to remain potent (30°C) (14).

### Day-28 Antibody Titre Comparison

**Figure 3:**
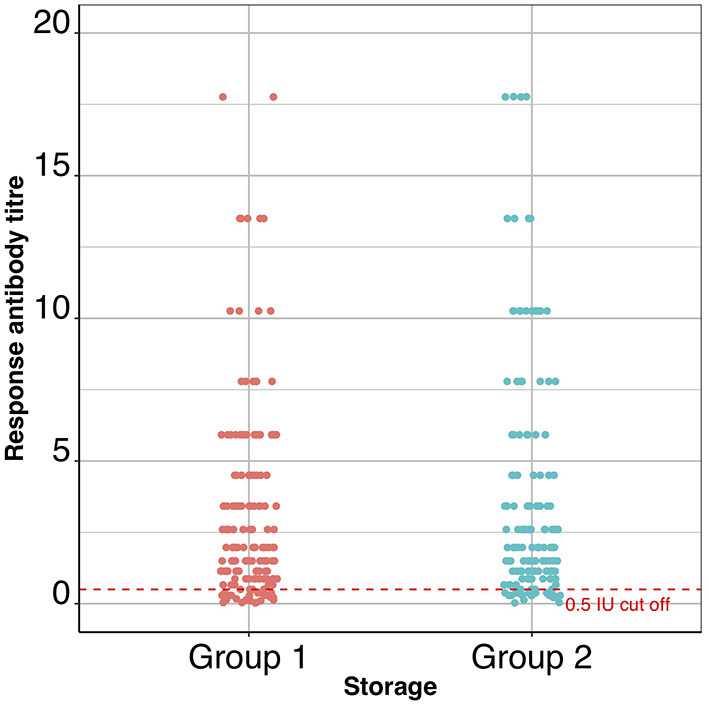
A dot-plot showing the titres (IU/ml) of all the dogs in the two groups of the trial. The dashed red line indicates the 0.5 IU/ml cut off point above which dogs are considered to have seroconverted.

The primary outcome variable was serological titre at day 28, and the predictor variable was the treatment group to which each dog belonged. The range of 28-day titres recorded for each group are shown in Figure 3 and the 28-day log_2_ and geometric mean titres are shown in Table 2. The output from the *T*-test examining the difference in titre levels between the cold-chain stored vaccine group (Group 1) and the elevated-temperature stored vaccine group (Group 2) was 0.18 with the lower 95% confidence limit (one-sided) of −0.17. Since the lower limit of the confidence interval did not exceed the non-inferiority −1.2 log_2_ titre units, the null hypothesis that non-cold-chain stored vaccine are less potent than cold-chain stored vaccine was rejected.

**Table 2:** Group summary data–the number of dogs in each group and the day-28 log mean, standard deviation, and geometric mean values are shown; day-28 log_2_ mean and day-28 SD represent the average and standard deviation of the log (base 2) transformed data for each group.

**Group**	**No. of dogs**	**Day-28 mean (log_**2**_)**	**Day-28 SD log_**2**_**	**Day-28 Geometric mean (IU/ml) log_**2**_**
1	163	0.9	2.0	1.8
2	163	1.0	1.9	2.0

The 28-day post vaccination group geometric mean titre was 1.8 and 2.0 IU/ml for cold-chain storage (Group 1) and non-cold-chain storage (Group 2), respectively.

A secondary outcome variable was the percentage of dogs that seroconverted in each group (Table 3), which were almost identical (~85%).

**Table 3:** Group seroconversion data–the number of dogs in each group, the number that seroconverted (≥0.5 IU/ml) and the seroconversion percentage are shown.

**Group**	**Total no. of dogs tested**	**No. of dogs seroconverted**	**% seroconversion**
1	163	139	85.3
2	163	140	85.7

### Exploratory Analysis: Impact of BCS and Age on Seroconversion at Day-28

**Figure 4:**
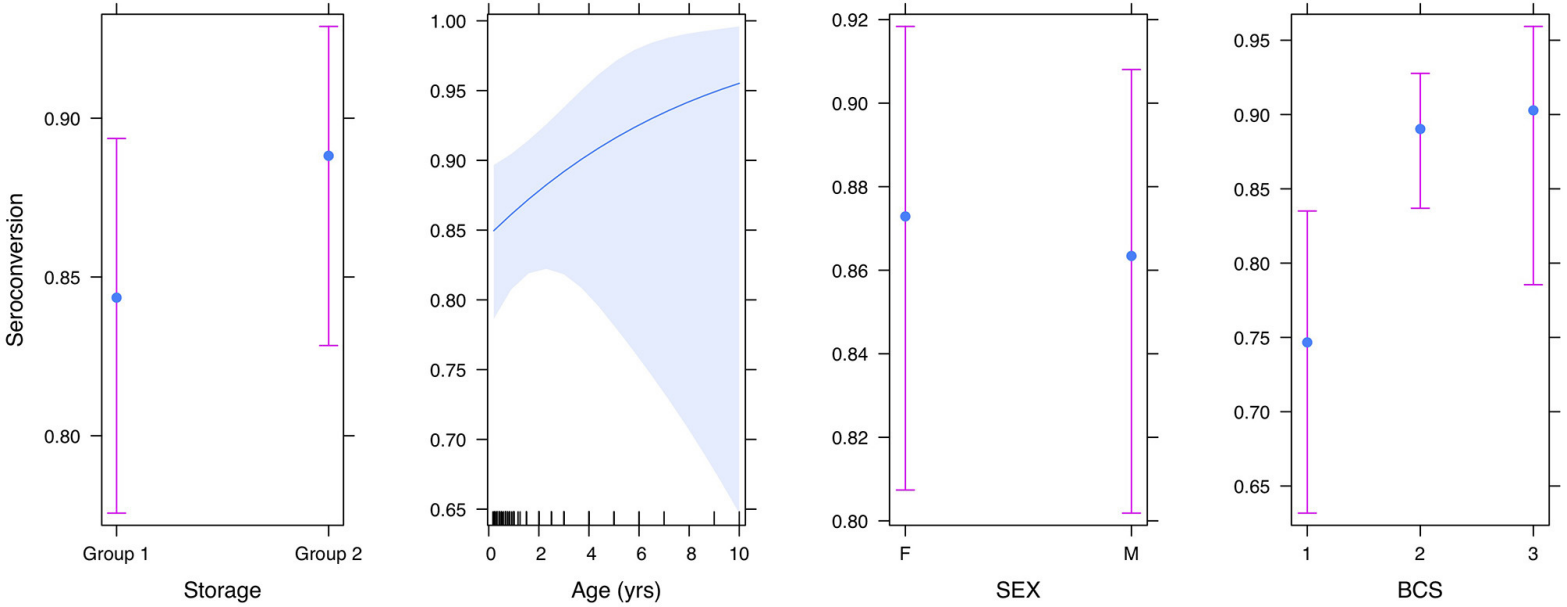
Plot showing the effect of storage condition, age, sex, and body condition score on seroconversion.

Logistic regression analyses were performed to examine the impact that BCS, sex, and age might have on the likelihood of seroconversion. The results (Table 4) indicated that, in corroboration of the *T*-test results, storage conditions did not affect the proportion of dogs that seroconverted (z = 1.1, df = 313, *p*-value = 0.25). There was a positive linear trend between BCS (O.R. 2.2, 95% CI: 1.1–5.1) and seroconversion (Figure 4), implying that as BCS increased so did the likelihood of seroconversion. Neither sex nor age impacted seroconversion.

**Table 4:** Multivariable logistic regression of a range of explanatory variables on the probability of seroconversion; Storage_Zeepot_ compares non-cold-chain storage with the baseline cold-chain storage; Age, age of dog; Sex_Male compares serological responses of male dogs with the baseline (female dogs); BCS, body condition score.

**Variable**	**Odds ratio**	**95% CI**	***P*-value**
Storage_Zeepot	1.5	0.7–2.9	0.25
Age	1.1	0.9–1.6	0.35
Sex_Male	0.9	0.5–1.8	0.81
BCS	2.2	1.1–5.1	0.04^*^

* *The p-value is significant at 0.05*.

## Discussion

This randomised controlled field trial demonstrates that the Nobivac^®^ Rabies vaccine stored within the Zeepot under fluctuating temperature conditions for up to 3 months elicited a neutralising antibody response equivalent to vaccine kept under stable cold-chain conditions. This conclusion is supported by the results from the logistic regression which showed storage condition had no effect on the seroconversion.

Following vaccination against rabies, dogs are considered protected when the immune system has elicited sufficient rabies virus-specific neutralising antibodies (RVSNA). RVSNA are a crucial component of adaptive immunity and contribute to the clearance of the viral infection (25). Globally, the antibody titre level of 0.5 IU/ml is recognised as the cut off above which humans, dogs and cats are classified as having developed a protective antibody response against rabies infection (24, 26). In this study, ~85% of the dogs both in the cold chain and non-cold chain group passed this threshold (Table 2). This proportion is slightly less than reported in the preceding study by Lankester et al. in which 92% of vaccinated dogs successfully seroconverted (14). A possible explanation for this reduction could be the health status of enrolled dogs. In the Lankester et al. study the experimental conditions of the trial were carefully controlled with the vaccine storage temperature kept stable, the age of enrolled dogs kept between 6 months and 5 years and the BCS between 2 and 4. In contrast, the conditions in this field study were purposively less stringent so that the trial reflected natural conditions more closely. As a result, storage temperatures were allowed to fluctuate and dogs were enrolled regardless of their BCS and age. This allowed investigation of the impact that age and BCS might have on sero-conversion and resulted in the finding that there was a statistically significant effect of BCS on the likelihood of seroconversion, with dogs of good body condition being more likely to seroconvert than dogs with poor BCS. This supports a previous study that suggested that loss of body condition was associated with immunosuppression (27). As such, it is possible that the reduction in the percentage of dogs that seroconverted was due to the inclusion of dogs of low BCS. Though the seroconversion was lower in dogs with low BCS it was still high; ~76% of dogs with BCS of 1 and 2 seroconverted. To improve seroconversion, a booster vaccination on day 28 could be given, but further investigation is needed to understand whether this is necessary.

The other factor that could have resulted in the lower seroconversion as compared to the Lankester et al. study was the fluctuating storage temperatures. But, as the percentage that seroconverted was the same for both treatment groups (cold-chain and non-cold-chain stored vaccine) this difference does not appear to reflect either an impact of non-cold-chain storage or temperature fluctuations. This finding is important as it indicates that the thermotolerance of the Nobivac Rabies vaccine is not dependent upon constant storage temperature conditions, as used in the Lankester et al. study, but rather is sustained despite the fluctuating storage temperatures as shown in Figure 2.

While the current study was not able to generate data on the longevity of the immune response in the non-cold chain group, previous studies have shown that when RVSNA are produced the immune memory B-cell immunity persists even after RVSNA drop below 0.1 IU/ml (28). Since both treatment groups elicited similar levels of antibody response, it seems unlikely that dogs vaccinated with the non-cold chain stored vaccine will have significantly shorter duration of immunity compared to those vaccinated with cold chain stored vaccine.

The multivariate analysis also demonstrated that sex had no effect on the seroconversion or titre. This result is consistent with findings from previous studies Berndtsson et al. (29) and Mansfield et al. (30).

The finding that the potency of the Nobivac vaccine was not impacted following storage in the Zeepot for the duration of 3 months has important implications for developing new immunisation strategies in remote or hard-to-reach areas facing logistical challenges in the transportation and storage of vaccine. As a result, decentralised storage strategies, involving vaccines being stored outside of cold-chain conditions for up to 3 months can be designed, implemented and tested for cost-effectiveness with the knowledge that such storage will not impact potency. Thermotolerance was a key factor that contributed to the successful campaigns to eradicate smallpox and rinderpest (31, 32). For example, with a shelf life of 8 months when kept at 37°C, it was possible to keep the rinderpest vaccine outside the cold chain for 30 days at a time, which gave sufficient time for vaccinators to travel on foot to implement vaccinations in extremely remote areas. This would not have been possible if conventional vaccine storage of 2–8°C was required. Likewise, the vaccination campaign against meningitis using the thermotolerant MenAfriVac's vaccine delivered in a controlled temperature device resulted in very high population coverage levels being achieved (11). The use of thermotolerant rabies vaccines stored without the need for electrical power in locally made Zeepots, outside of cold chain conditions, could bring similar benefits, in coverage and cost-effectiveness, to the control of dog-mediated human rabies.

## Conclusion

The results obtained in this study clearly demonstrated that the Nobivac^®^ Rabies vaccine kept in locally made Zeepots elicited protective neutralising antibody responses equivalent to those from vaccine stored under cold chain conditions. These findings will help in the design of cost-effective vaccine delivery strategies for use in resource-poor settings and will be of benefit to nations designing national elimination plans in an attempt to hit the international commitment of zero human deaths by 2030, “Zero by 30” (33).

## Data Availability Statement

The raw data supporting the conclusions of this article will be made available by the authors, without undue reservation.

## Ethics Statement

The animal study was reviewed and approved by Institutional Animal Care and Use Committee, Washington State University and Sokoine University of Agriculture. Written informed consent was obtained from the owners for the participation of their animals in this study.

## Author Contributions

AL, KH, and FL made substantial contributions to the study design. AL, AC, and MB collected the data. AL, FL, KH, LM, and DM analysed and interpreted the data. AL led the writing of the manuscript, which all authors contributed to. AL, FL, RK, and KH contributed to reviewing and proof-reading the manuscript as well as interpretation and synthesis. All authors read and approved the version of the manuscript to be published.

## Funding

This research was funded by the Department of Health and Human Services of the National Institutes of Health under grant number R01AI141712 and MSD Animal Health provided the vaccines free of charge. Additionally, AL was supported by the DELTAS Africa Initiative (Afrique One-ASPIRE/DEL-15-008). Afrique One-ASPIRE was funded by a consortium of donors including the African Academy of Sciences (AAS) Alliance for Accelerating Excellence in Science in Africa (AESA), the New Partnership for Africa's Development Planning and Coordinating (NEPAD) Agency, the Wellcome Trust (107753/A/15/Z), and the UK government. KH was funded through the Wellcome Trust (207569/Z/17/Z). LM and DM were supported by Defra, the Scottish Government, and the Welsh Government via grant SV3500.

## Author Disclaimer

The content is solely the responsibility of the authors and does not necessarily represent the official views of the National Institutes of Health.

## Conflict of Interest

The authors declare that the research was conducted in the absence of any commercial or financial relationships that could be construed as a potential conflict of interest.

## Publisher's Note

All claims expressed in this article are solely those of the authors and do not necessarily represent those of their affiliated organizations, or those of the publisher, the editors and the reviewers. Any product that may be evaluated in this article, or claim that may be made by its manufacturer, is not guaranteed or endorsed by the publisher.
